# Genetic variants of increased waist circumference in psychosis

**DOI:** 10.1097/YPG.0000000000000181

**Published:** 2017-10-26

**Authors:** Dzana S. Hukic, Urban Ösby, Eric Olsson, Agneta Hilding, Claes-Göran Östenson, Harvest F. Gu, Ewa Ehrenborg, Gunnar Edman, Martin Schalling, Catharina Lavebratt, Louise Frisén

**Affiliations:** Departments of a Molecular Medicine and Surgery, Neurogenetics Unit; b Neurobiology, Care Sciences and Society, Centre for Family Medicine; c Clinical Neuroscience; d Molecular Medicine and Surgery, Endocrine and Diabetes Unit; e Medicine, Cardiovascular Medicine Unit, Karolinska Institutet; f Center for Molecular Medicine, Karolinska University Hospital Solna; g Department of Adult Psychiatry, PRIMA Barn och Vuxenpsykiatri AB; h Child and Adolescent Psychiatry Research Center, Stockholm, Sweden

**Keywords:** association study, case–case, case–control, diabetes mellitus type 2, metabolic risk genes, psychotic disorder

## Abstract

We examined whether established metabolic risk genetic variants in the population confer a risk for increased waist circumference in patients with schizophrenia spectrum disorders and also an association with schizophrenia spectrum disorders irrespective of waist circumference.

**Patients and methods:**

We analyzed the association in (i) a case–case model in which patients with schizophrenia spectrum disorder with increased waist circumference (≥80 cm for women and ≥94 cm for men) (*n*=534) were compared with patients with normal waist circumference (<80 cm for women; <94 cm for men) (*n*=124), and in (ii) a case–control model in which schizophrenia spectrum disorder patients with increased waist circumference or irrespective of waist circumference were compared with population-derived controls (*n*=494) adjusted for age, sex, fasting glucose, smoking, and family history of diabetes.

**Results:**

Genetic variants in five genes (*MIA3*, *MRAS*, *P2RX7*, *CAMKK2*, and *SMAD3*) were associated with increased waist circumference in patients with schizophrenia spectrum disorder (*P*<0.046). Genetic variants in three other genes (*PPARD*, *MNTR1B*, and *NOTCH2*) were associated with increased waist circumference in patients when compared with control individuals (*P*<0.037). Genetic variants in the *PPARD*, *MNTR1B*, *NOTCH2*, and *HNF1B* were nominally associated with schizophrenia spectrum disorder irrespective of waist circumference (*P*<0.027). No differences in waist circumference between specific psychosis diagnoses were detected.

**Conclusion:**

Increased waist circumference in patients with schizophrenia spectrum disorder may be explained, in part, by increased metabolic risk gene burden, and it indicates a shared genetic susceptibility to metabolic disorder and psychosis *per se*. Along these lines, common metabolic risk genetic variants confer a risk for increased waist circumference in patients with schizophrenia spectrum disorders.

## Introduction

An increased risk of metabolic disturbances in patients with severe mental illness, including obesity and diabetes mellitus type 2, is well documented in a large number of studies. A doubled rate of mortality from cardiovascular disease has been repeatedly shown (Osby *et al.*, 2000; Hennekens *et al.*, 2005; Gothefors *et al.*, 2010). The increased mortality from cardiovascular disease is of great clinical importance because it is the main cause of death leading to reduced life expectancy in severe mental illness (Laursen *et al.*, 2013; Nordentoft *et al.*, 2013). Thus, further knowledge about the mechanisms of cardiovascular disease in severe mental illness is strongly warranted.

Antipsychotic medication, especially atypical antipsychotics, is well known to cause weight gain, but other factors also contribute. Studies of drug-naive patients and drug-free patients indicate increased levels of visceral fat deposition (Thakore *et al.*, 2002), supporting the view that psychotic disorder *per se* is linked to metabolic disturbances (Osby *et al.*, 2013). In the population, there is a substantial genetic vulnerability for increased body weight and increased waist circumference. Increased waist circumference is the established measure of central obesity, that is, excess adipose tissue (Pouliot *et al.*, 1994; Janssen *et al.*, 2004), and high values predispose for metabolic disorders irrespective of weight (Despres and Lemieux, 2006; Romero-Corral *et al.*, 2010; Phillips, 2013). Thus, analysis of the genetic contribution to metabolic disturbances in severe mental illness patients measured by waist circumference might therefore be of substantial clinical interest.

The purpose of this study was to (i) investigate whether common metabolic genetic variants confer a risk for increased waist circumference in patients with schizophrenia spectrum disorders (SSD), and to (ii) investigate the genetic variants linked to SSD, irrespective of waist circumference.

## Patients and methods

### Ethical approval

Ethical approval was obtained from the Stockholm Regional Ethics Committee separately for patients and controls. All participants gave their informed consent to participate.

### Patients from the Swedish study of metabolic risks in psychosis

Patients were recruited from specialized psychosis outpatient clinics, primarily in Stockholm County, Sweden, responsible for treatment of patients with long-term psychotic disorders, especially schizophrenia, between 2005 and 2009. As part of a general medical examination, all patients were asked to participate in the Swedish study of metabolic risks in psychosis (SMRP). Patients received written instructions to fast overnight before venous blood sampling. Fasting glucose, blood pressure, body weight, height, and waist circumference were measured. Patients were asked about tobacco and alcohol use, family history of diabetes, and medications and dosage. Clinical diagnoses were confirmed according to the *Diagnostic and Statistical Manual of Mental disorders*, Washington DC. American Psychiatric Association, 4th ed. (1994). In the present study of severe mental illness patients in clinical treatment, 658 SSD patients were included, with schizophrenia being the most common diagnosis for 356 (54%) patients, schizoaffective disorder for 68 (10%) patients, delusional disorder for 41 (6%) patients, psychosis not otherwise specified for 88 (14%) patients, bipolar disorder for 40 (6%) patients, and other psychiatric disorders for 65 (10%) patients.

### Stockholm Diabetes Prevention Program controls

Control individuals were selected from the Stockholm Diabetes Prevention Program (SDPP) (Eriksson *et al.*, 2008), comprising 7949 participants included from 1992 to 1998. At inclusion, only patients without known diabetes were enrolled and half of the patients had at least 1 first-degree relative with known diabetes. A follow-up was performed 9–10 years later (2002–2006) and included 5712 patients (3329 women and 2383 men) (72% of the original participants). At follow-up, 997 (17%) individuals had increased fasting glucose levels (≥5.6 mmol/l), including 289 (5%) individuals who were diagnosed with type 2 diabetes during the period between inclusion and follow-up. Data were obtained about weight, height, waist circumference, blood pressure, and fasting blood glucose both at inclusion and at follow-up.

### Stockholm Diabetes Prevention Program Genetic controls

From the SDPP follow-up sample, 494 controls were selected to represent the total SDPP cohort for the genetic association study. In the control group, 404 (82%) patients had normal fasting glucose levels, 66 (13%) patients had increased fasting glucose levels, 24 (5%) patients were diagnosed with type 2 diabetes, and 185 (37%) had a family history of diabetes. Furthermore, 147 (30%) patients had normal waist circumference levels, and 347 (70%) patients had increased waist circumference (Table 1).

**Table 1:**
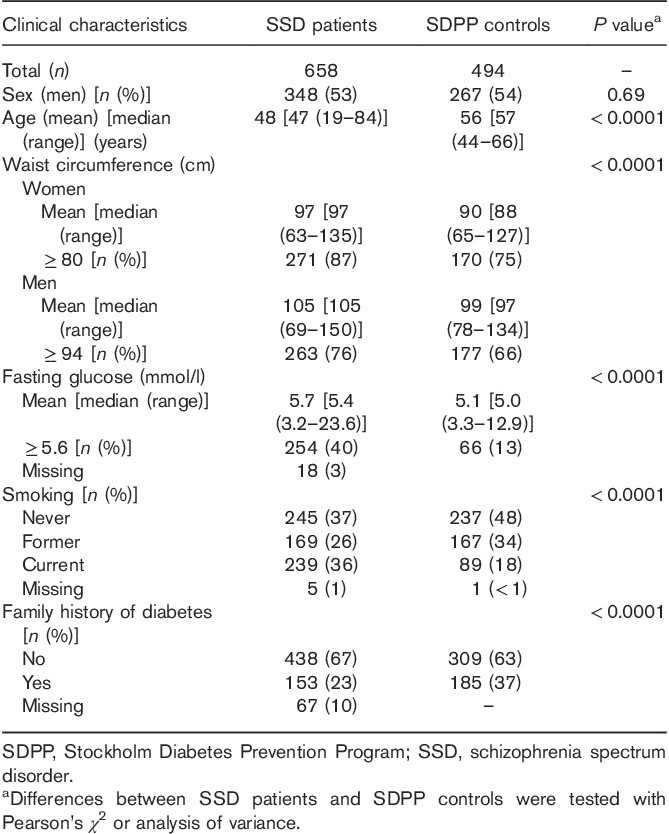
Clinical characteristics of the patients and controls

### DNA preparation and genotyping for the patients and Stockholm Diabetes Prevention Program controls

DNA from venous blood was extracted according to standard procedures. Single-nucleotide polymorphisms (SNPs) were genotyped using an Open Array Real-Time PCR System Instrument (Applied Biosystems, Foster City, California, USA). Allelic discrimination was performed using TaqMan Genotype Software (Applied Biosystems). Genotyping success rates for the SNPs were between 85 and 98%.

### Genetic variants assessed

SNPs previously reported to be associated with type 2 diabetes and/or cardiovascular disease were studied. The majority of these SNPs (25 of 46) showed genome-wide significance (*P*<5×10^−8^) in Genome-Wide Association (GWA) studies (Table 2). Hardy–Weinberg equilibrium cutoff was *P* up to 0.05 for controls.

**Table 2:**
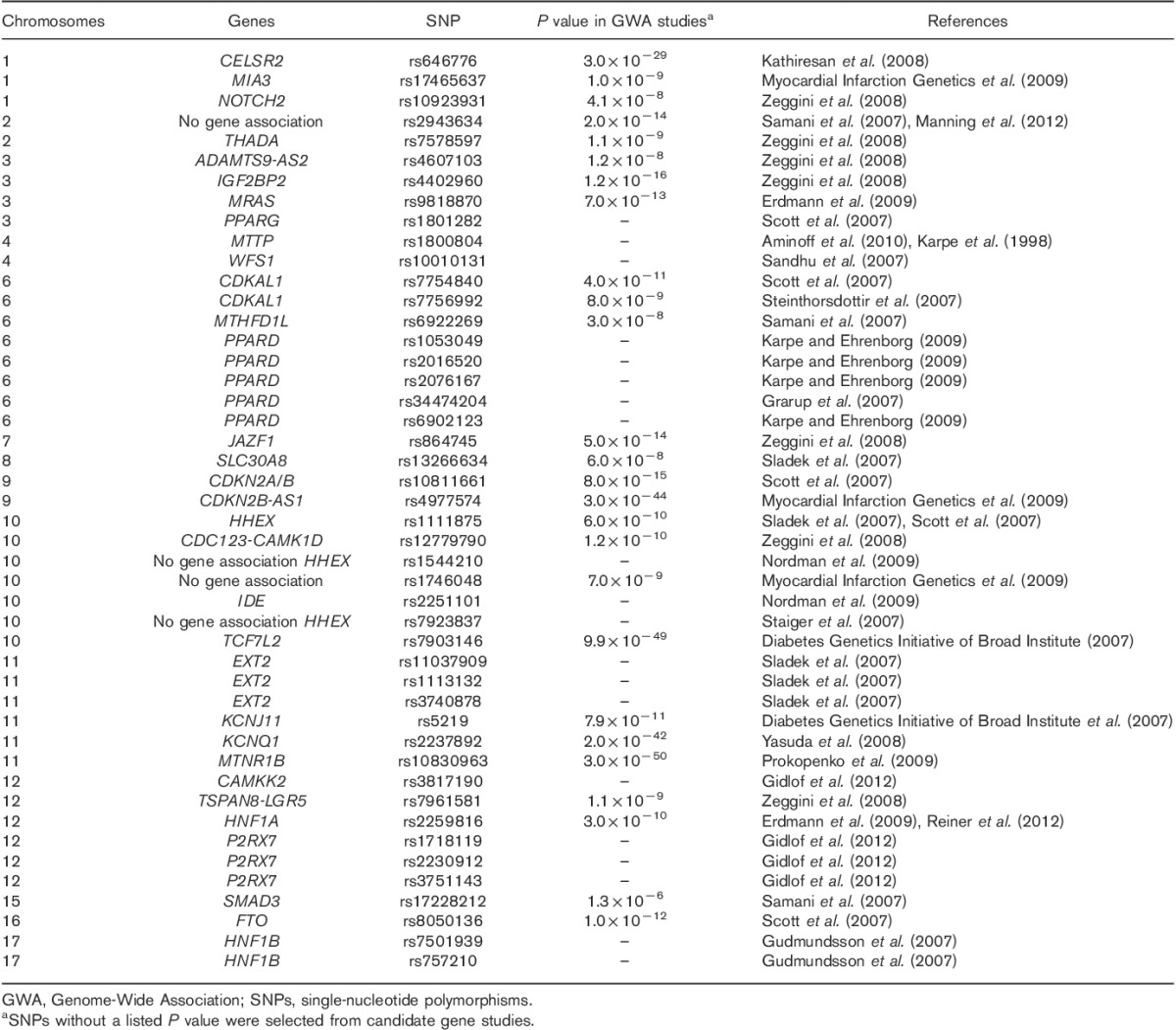
Single-nucleotide polymorphisms studied

### Study design and statistical analyses

Central obesity was measured by increased waist circumference. The cutoff level for increased waist circumference (≥80 cm women and ≥94 cm men) was defined according to criteria from the International Diabetes Federation (*http://www.idf.org/webdata/docs/Metabolic_syndrome definition.pdf*).

Before performing genetic analysis, differences in waist circumference between psychosis diagnoses were analyzed, separately for men and women. First, differences in waist circumference between psychosis diagnoses, for each sex, were tested for significance using analysis of variance in IBM SPSS Statistics 23 (IBM Corporation, Armonk, New York, USA).

Second, patients were analyzed for allelic association to the listed SNPs according to three models: model 1, a case–case model in which SSD patients with increased waist circumference (≥80 cm for women, ≥94 for men) were compared with SSD patients with normal waist circumference (<80 cm women, <94 cm men); model 2, a case–control model in which SSD patients with increased waist circumference were compared with SDPP controls; and model 3, another case–control model in which all SSD patients were compared with SDPP controls. Logistic regression was used in models 1 and 2, adjusted for the continuous variables age and fasting glucose, and the categorical variables family history of diabetes, sex, and smoking. The logistic regression in model 3 was adjusted for the same factors and also the categorical variable waist circumference. For multiple testing correction, false discovery rate Benjamini and Hochberg was used.

Third, to test the effect of clozapine treatment on nominal allelic associations with increased waist circumference, analyses with models 1 and 2 were performed where SSD cases were restricted to patients on clozapine (*n*=62).

The allelic association analyses were performed using PLINK (Center for Human Genetic Research, Massachusetts General Hospital, Boston, Massachusetts, USA; *http://pngu.mgh.harvard.edu/purcell/plink/*) (Purcell *et al.*, 2007). The level of nominal significance was set to 5% (two tailed).

## Results

### Genetic findings

No differences in waist circumference were detected (*P*>0.05) between psychosis diagnoses. All SNPs except rs864745, rs12779790, rs2251101, and rs2016520 (excluded from analysis) were in Hardy–Weinberg equilibrium (*P*>0.05). In the case–case design, increased waist circumference was associated with SNPs located within *MIA3*, *MRAS*, *P2RX7*, *CAMKK2*, and *SMAD3*, and in the case–control design with SNPs in *PPARD*, *MTNR1B*, *NOTCH2*, and *HNF1B* (Table 3).

**Table 3:**
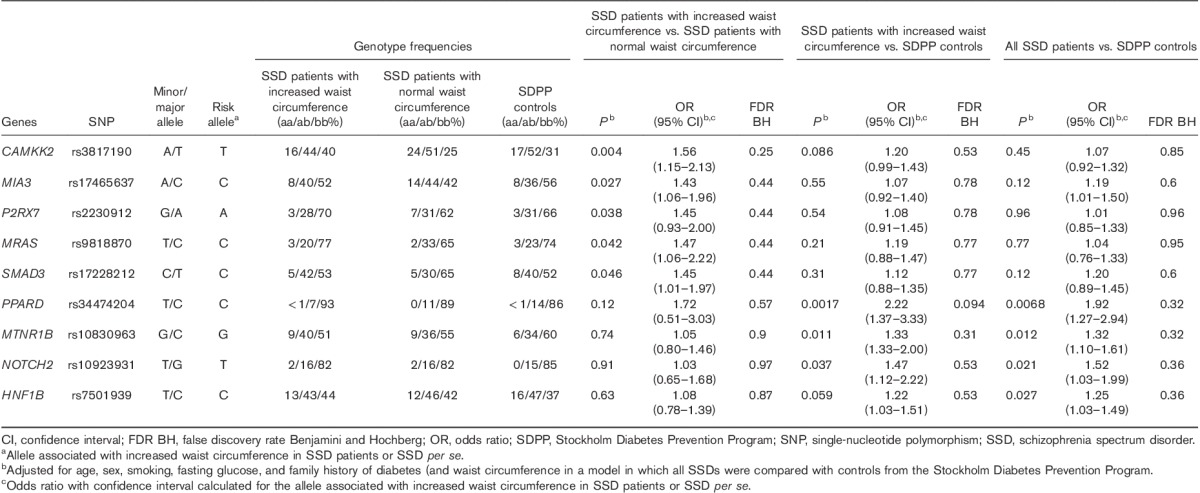
Allelic association between metabolic risk variants and increased waist circumference in schizophrenia spectrum disorder, and schizophrenia spectrum disorder *per se*

### Case–case design

In the case–case analysis, the major allele T of rs3817190 in *CAMKK2* [odds ratio (OR): 1.56, *P*=0.0040), the major allele C of rs17465637 (OR: 1.43, *P*=0.027) in *MIA3*, the major allele A of rs2230912 (OR: 1.45, *P*=0.038) in *P2RX7*, and the major allele C of rs9818870 (OR: 1.47, *P*=0.042) in *MRAS* were nominally associated with increased waist circumference among SSD patients compared with the minor allele. The minor allele C of rs17228212 in *SMAD3* (OR: 1.45, *P*=0.046) was associated with increased waist circumference compared with the major allele.

### Case–control design

In the case–control analysis, the major allele C of rs34474204 (OR: 2.22, *P*=0.0017) in *PPARD*, the minor allele G of rs10830963 (OR: 1.33, *P*=0.011) in *MNTR1B*, and the minor allele T of rs10923931 (OR: 1.47, *P*=0.037) in *NOTCH2* were nominally associated with increased waist circumference among the SSD patients compared with the other allele (Table 2).

### Genetic variants associated with schizophrenia spectrum disorder *per se* (irrespective of waist circumference)

In the allelic analysis of all SSD patients (*n*=658) compared with controls, the major allele C of rs34474204 (OR: 1.92, *P*=0.0068) in *PPARD*, the minor allele G of rs10830963 (OR: 1.32, *P*=0.012) in *MTNR1B*, the minor allele T of rs10923931 (OR: 1.52, *P*=0.021) in *NOTCH2*, and the major allele C of rs7501939 (OR: 1.25, *P*=0.027) in *HNF1B* were associated with SSD patients irrespective of waist circumference. The nine SNPs, with a signal in this study, together as predictors accounted for 1% (adjusted *R*^2^: 0.001) of the variance in psychosis *per se*.

### The effect of clozapine treatment on allelic associations with increased waist circumference

To test the effect of clozapine treatment on nominal allelic associations with increased waist circumference, the ORs were calculated including only psychosis patients on clozapine treatment. The point estimates of ORs were well within the 95% confidence intervals (CIs)of ORs based on patients irrespectively of pharmacotherapy, except for *NOTCH2* (Table 4).

**Table 4:**
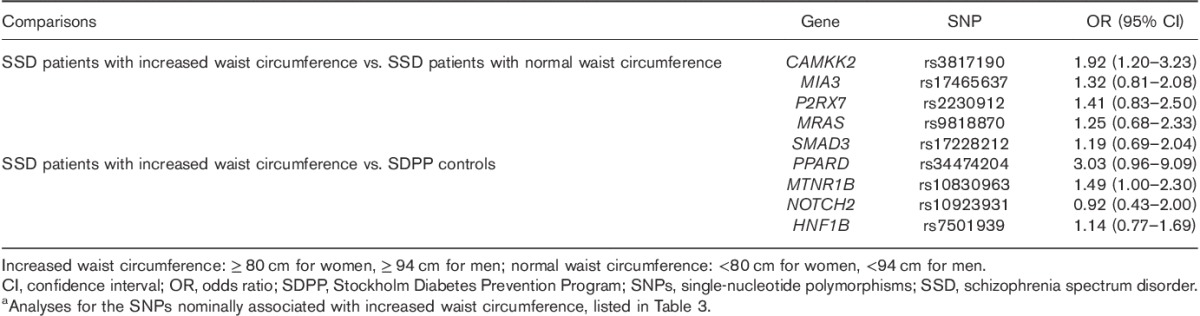
Odds ratios for risk alleles in relation to disorder status in schizophrenia spectrum disorder patients on clozapine treatment^a^ (*n*=62)

## Discussion

### Main findings

The findings of this study were that increased waist circumference in patients with SSD was associated with common metabolic genetic variants in *MIA3*, *MRAS*, *P2RX7*, *CAMKK2*, and *SMAD3* when compared with SSD patients with normal waist circumference, and with genetic variants in the *PPARD*, *MNTR1B*, and *NOTCH2* in comparison with control individuals. The genetic variants in *PPARD, MNTR1B*, *NOTCH2*, and *HNF1B* were nominally associated with SSD *per se*.

### Strengths and limitations

In the present study, we applied both a case–case and a case–control design. A case–case model takes advantage of narrow diagnostic subgroups considered to be more biologically homogeneous, and therefore may imply clinical heterogeneity differences between disease groups (Niculescu and Le-Niculescu, 2010).

At the decade around sampling, there were no solid internal genetic limits in the Swedish population, particularly the southern/middle parts of Sweden (the recruitment areas) were more genetically homogeneous (Lappalainen *et al.*, 2009; Humphreys *et al.*, 2011). The patient sample was recruited from specialized psychosis outpatient clinics. Participation rate was estimated from one clinic: 119 of the 155 (77%) patients participated. There was no difference in BMI between those who participated and those who declined. As waist circumference did not differ between different psychosis diagnoses, SSD patients were analyzed as one group; thus, findings from the present study may be generally applicable to increased waist circumference in SSD patients.

For the genetic association study, SDPP controls were selected in the interest of representing the whole SDPP sample at follow-up. However, at inclusion, only individuals without known diabetes were enrolled, and there was a high frequency of family history of diabetes among them.

Family history of diabetes, age, fasting glucose, and smoking may influence the risk for diabetes mellitus type 2, and we were able to adjust for these differences. As only patients, and no controls, had antipsychotic medication, this was not possible to control for. Instead, we tested the differential effect of clozapine, the antipsychotic drug associated with the greatest weight gain, previously evaluated in our patient sample (Boden *et al.*, 2013). The low statistical power indicates a possible risk that we were not able to detect true genetic associations between the groups that may exist. No associations survived correction for multiple testing using false discovery rate assessment of the entire data set. However, given that each SNP was individually selected on the basis of previously published functionality and association with metabolic disorder, this correction might be regarded as overstringent.

### Findings from other studies

Waist circumference is a convenient measure of excess adipose tissue and central obesity (Pouliot *et al.*, 1994; Janssen *et al.*, 2004). Excessive amounts of body adipose tissue predispose to metabolic disorders, irrespective of weight (Despres and Lemieux, 2006; Romero-Corral *et al.*, 2010; Phillips, 2013). Persons with a family history of diabetes generally have an increased storage of fat and risk for obesity, as well as a decreased beta cell function, compared with persons without family history of diabetes (Hilding *et al.*, 2006; Isomaa *et al.*, 2010), which is in agreement with a genetic component in metabolic disorders. Similar metabolic disturbances have been observed in psychosis patients, although antipsychotics have been associated with weight gain. An increased prevalence of diabetes mellitus type 2 has been reported in first-episode drug-naive psychosis patients (Ryan *et al.*, 2003), and unaffected first-degree relatives of people with schizophrenia (Fernandez-Egea *et al.*, 2008). Psychotic disorder *per se* increases the risk for elevated waist circumference and fasting glucose (Osby *et al.*, 2013).

In this report, metabolic gene variants known to increase the risk for metabolic disorders in the population also seem to confer metabolic risk in SSD patients. The genetic variants rs17465637 in *MIA3*, rs9818870 in *MRAS*, and rs17228212 in *SMAD3* have been associated with coronary artery disease in GWA studies (*P*<5×10^−8^), although explaining only a small proportion of the coronary artery disease risk (Samani *et al.*, 2007; Erdmann *et al.*, 2009). However, for rs9818870 in *MRAS* we identified the other allele as a risk allele among SSD patients, in contrast to previous findings. The *MRAS* gene encodes a protein that functions as a signal transducer in, for example, cell growth and differentiation. *SMAD3* and *MIA3* are important for vascular stability (Itoh *et al.*, 2012) and angiogenesis (Bosserhoff and Buettner, 2002), fundamental processes for plaque development and atherosclerosis, although the mechanisms are not fully known. *SMAD3* mediates transcription activity downstream of TGFB and *MIA3* encodes a translation factor. The *P2RX7* gene encodes an ATP-binding receptor calcium channel protein that mediates apoptosis, and its activation may also lead to changes in gene expression. *CAMKK2* encodes a protein kinase that responds to increased intracellular calcium, and one of its many functions is to regulate the production of the appetite-stimulating hormone neuropeptide Y. *P2RX7* and *CAMKK2* have been associated with a decreased risk of cardiovascular events (Gidlof *et al.*, 2012). In addition to metabolic disturbances, *P2RX7* has also been implicated in psychiatric disorders (Backlund *et al.*, 2012). The reported risk alleles of genetic variants rs10830963 in *MTNR1B* and rs10923931 in *NOTCH2* were associated with diabetes mellitus type 2-related traits in GWA studies (*P*<5×10^−8^) (Zeggini *et al.*, 2008; Prokopenko *et al.*, 2009). We here suggest that the same risk alleles are associated with diabetes mellitus type 2-related traits among SSD patients. *MNTR1B* encodes a G-protein-coupled membrane protein whose variants are well known to affect fasting glucose levels. β-Cells from diabetic patients, similar to nondiabetic individuals, carrying the risk allele G of the rs10830963 have increased *MTNR1B* receptor expression, supported by altered insulin release in the presence of melatonin (Lyssenko *et al.*, 2009). Recently, we reported an association for the rs10830963 allele G (OR: 1.51, 95% CI: 1.16–1.89; *P*=0.0039) and rs10923931 allele T (OR: 1.84, 95% CI: 1.13–2.46; *P*=0.011) to increased fasting glucose levels in SSD patients (Hukic *et al.*, 2015). In addition to metabolic traits, Notch signaling has been shown to be important for neurogenesis in adult brain (Ables *et al.*, 2011), and has been implicated in subphenotypes of psychiatric disorders (Prox *et al.*, 2013; Monsalve *et al.*, 2014). The *NOTCH2* gene encodes a transmembrane protein that regulates interactions between physically adjacent cells. Common genetic variants in *PPARD* in relation to metabolic traits have generated conflicting results (Grarup *et al.*, 2007). However, *PPARD* has been associated with glucose metabolism and function (Kramer *et al.*, 2005; Karpe and Ehrenborg, 2009). The *PPARD* gene encodes a nuclear hormone receptor that may function as an integrator of transcription repression and nuclear receptor signaling. Changes in expression of the *PPARD* gene have been reported in newly diagnosed diabetics (Stoynev *et al.*, 2014). The *HNF1B* gene encodes transcription factor 2, a liver-specific factor of the homeobox-containing basic helix-turn-helix family that may activate or inhibit transcription of target genes. Although it has primarily been associated with prostate cancer, mutations in this gene have been identified as the cause of maturity-onset of diabetes type 5. Here we report an association with schizophrenia *per se*.

### The effect of clozapine treatment on the allelic associations with increased waist circumference

Treatment with clozapine is known to be associated with prominent weight gain. To explore the effect of clozapine on our nominal allelic associations reported here, analyses restricted to patients on clozapine were performed. These analyses resulted in pointwise ORs within the 95% CIs for all markers and model designs, except for *NOTCH2*, suggesting that the effect sizes of clozapine on observed genetic associations with increased waist circumference were limited, although *NOTCH2* pointed the contrary, suggesting the other allele as a risk allele for SSD patients on clozapine treatment. Analysis of larger samples, as well as in drug-naive samples, is warranted. The severity of weight gain during treatment is also correlated with the initial weight; however, this is a cross-sectional patient group in which weight gain associated with specific antipsychotic drugs was not possible to measure.

The findings of the present study of associations between genes conferring increased metabolic risk and increased waist circumference in patients with SSD indicate that increased waist circumference in those patients may be explained, in part, by an increased genetic vulnerability for metabolic risk genes, and indicates a shared genetic susceptibility to metabolic disorder and psychosis *per se*. All findings indicating factors that increase the metabolic risk, for example, increasing waist circumference, in SSD patients might be of clinical interest, as increased morbidity and mortality from cardiovascular disorders is the main cause of the reduced longevity of patients with psychosis.

Our results might propose shared genetic susceptibility to metabolic disorder and psychosis *per se*, but replication using a polygenic risk scoring approach in GWAS data, by using SNPs linked to metabolic disturbances on a psychosis versus healthy controls data set, is required for verification.
